# 2-[(*R*)-Hydr­oxy(6-methoxy­quinolinium-4-yl)meth­yl]-8-vinyl-1-azoniabicyclo­[2.2.2]octane tetra­chloridoferrate(III) chloride monohydrate

**DOI:** 10.1107/S1600536810007889

**Published:** 2010-03-06

**Authors:** Li-Zhuang Chen, Mei-Na Huang

**Affiliations:** a School of Material Science and Engineering, Jiangsu University of Science and Technology, Zhenjiang 212003, People’s Republic of China

## Abstract

In the title salt, (C_20_H_26_N_2_O_2_)[FeCl_4_]Cl·H_2_O, the Fe^III^ atom exists in a tetra­hedral coordination environment. The cation, anions and water mol­ecules are linked by N—H⋯Cl, O—H⋯Cl and O—H⋯O hydrogen bonds into a layer network.

## Related literature

For ferroelectricity and SHG of chiral coordination compounds, see: Fu *et al.* (2007 ▶); Qu *et al.* (2003 ▶). For related transition-metal complexes, see: Zhao *et al.* (2003 ▶).
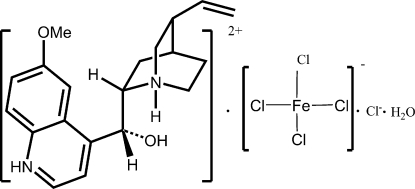


## Experimental

### 

#### Crystal data

(C_20_H_26_N_2_O_2_)[FeCl_4_]Cl·H_2_O
*M* *_r_* = 577.54
Monoclinic, 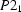

*a* = 6.6838 (10) Å
*b* = 18.843 (2) Å
*c* = 10.8716 (10) Å
β = 104.918 (17)°
*V* = 1323.1 (3) Å^3^
*Z* = 2
Mo *K*α radiation
μ = 1.10 mm^−1^
*T* = 293 K
0.30 × 0.26 × 0.22 mm

#### Data collection

Rigaku SCXmini diffractometer
Absorption correction: multi-scan (*CrystalClear*; Rigaku, 2005 ▶) *T*_min_ = 0.82, *T*_max_ = 0.88
12145 measured reflections
5166 independent reflections
3650 reflections with *I* > 2σ(*I*)
*R*_int_ = 0.038

#### Refinement

*R*[*F* ^2^ > 2σ(*F* ^2^)] = 0.048
*wR*(*F* ^2^) = 0.118
*S* = 1.01
5166 reflections
281 parameters
1 restraint
H-atom parameters constrained
Δρ_max_ = 0.32 e Å^−3^
Δρ_min_ = −0.30 e Å^−3^
Absolute structure: Flack (1983 ▶), 2490 Friedel pairs
Flack parameter: 0.01 (2)

### 

Data collection: *CrystalClear* (Rigaku, 2005 ▶); cell refinement: *CrystalClear*; data reduction: *CrystalClear*; program(s) used to solve structure: *SHELXS97* (Sheldrick, 2008 ▶); program(s) used to refine structure: *SHELXL97* (Sheldrick, 2008 ▶); molecular graphics: *SHELXTL* (Sheldrick, 2008 ▶); software used to prepare material for publication: *SHELXL97*.

## Supplementary Material

Crystal structure: contains datablocks I, global. DOI: 10.1107/S1600536810007889/ng2725sup1.cif

Structure factors: contains datablocks I. DOI: 10.1107/S1600536810007889/ng2725Isup2.hkl

Additional supplementary materials:  crystallographic information; 3D view; checkCIF report

## Figures and Tables

**Table 1:** Hydrogen-bond geometry (Å, °)

*D*—H⋯*A*	*D*—H	H⋯*A*	*D*⋯*A*	*D*—H⋯*A*
N1—H1*B*⋯Cl5	0.96	2.10	3.023 (4)	161
N2—H2*C*⋯Cl5^i^	0.96	2.08	3.039 (4)	173
O2—H2*B*⋯O3	0.85	2.00	2.799 (6)	156
O3—H3*B*⋯Cl5^ii^	0.85	2.71	3.070 (6)	108

Symmetry codes: (i) 
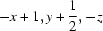
; (ii) 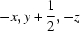.

**Fig. 1.**
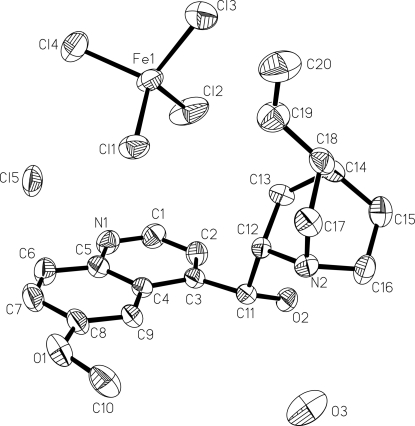
The asymmetric unit of the title compound with atom labels. Displacement ellipsoids were drawn at the 30% probability level. Hydrogen atoms have been omitted for clarity.

**Fig. 2.**
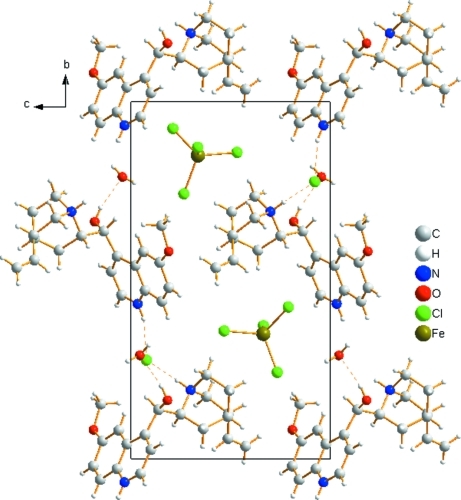
The packing viewed along the c axis. Hydrogen bonds are drawn as dashed lines

## supplementary crystallographic information

### Comment

The existence of a chiral centre in an organic ligand is very important for the construction noncentrosymmetric or chiral coordination polymers that exhibit desirable physical properties such as ferroelectricity (Fu *et al.*, 2007), Chiral quinine has a chiral centre which have shown tremendous scope in the synthesis of transition-metal complexes (Zhao *et al.*, 2003; Qu *et al.*,2003). The construction of new members of this family of ligands is an important direction in the development of modern coordination chemistry. We report here the crystal structure of the title compound

The asymmetric unit of the title compound,*C*_20_H_26_N_2_O_2_.FeCl_4_.Cl.H_2_O(Fig.1), consists of one protoned quinine and a tetrachloro-ironanion with the Fe^III^ ion in a slightly distorted tetrahedral coordination environment, The crystal structure is stabilized by intermolecular N—H···Cl, O—H···Cl and O—H···O hydrogen bonds.The H-bonds form of1D chain viewedalong the *a*-axis (Fig.2).

### Experimental

A mixture of quinine (1 mmol, 0.324 g ), FeCl_3_(1 mmol, 0.156 g) and 10% aqueous HCl (6 ml) were mixed and dissolved in 20 ml water by heating to 353 K (0.5 h) forming a clear solution. The reaction mixture was cooled slowly to room temperature, crystals of the title compound were formed after 11 days.

### Refinement

All H atoms of quinine were placed in calculated positions , with C—H = 0.93-0.98 Å O—H = 0.85 Å and N—H = 0.96 Å, and re?ned using a riding model, with U_iso_(H)=1.2U_eq_(C, N, O) or 1.5 U_eq_(C) for methyl H atoms.H3A and H3B were located in difference fourier maps.

### Crystal data

**Table:**

(C_20_H_26_N_2_O_2_)[FeCl_4_]Cl·H_2_O	*F*(000) = 594
*M**_r_* = 577.54	*D*_x_ = 1.450 Mg m^−^^3^
Monoclinic, *P*2_1_	Mo *K*α radiation, λ = 0.71073 Å
Hall symbol: P 2yb	Cell parameters from 3650 reflections
*a* = 6.6838 (10) Å	θ = 2.9–26.0°
*b* = 18.843 (2) Å	µ = 1.10 mm^−^^1^
*c* = 10.8716 (10) Å	*T* = 293 K
β = 104.918 (17)°	Block, yellow
*V* = 1323.1 (3) Å^3^	0.30 × 0.26 × 0.22 mm
*Z* = 2	

### Data collection

**Table:**

Rigaku SCXmini diffractometer	5166 independent reflections
Radiation source: fine-focus sealed tube	3650 reflections with *I* > 2σ(*I*)
graphite	*R*_int_ = 0.038
Detector resolution: 13.6612 pixels mm^-1^	θ_max_ = 26.0°, θ_min_ = 2.9°
ω scans	*h* = −8→8
Absorption correction: multi-scan (*CrystalClear*; Rigaku, 2005)	*k* = −23→23
*T*_min_ = 0.82, *T*_max_ = 0.88	*l* = −13→13
12145 measured reflections	

### Refinement

**Table:**

Refinement on *F*^2^	Secondary atom site location: difference Fourier map
Least-squares matrix: full	Hydrogen site location: inferred from neighbouring sites
*R*[*F*^2^ > 2σ(*F*^2^)] = 0.048	H-atom parameters constrained
*wR*(*F*^2^) = 0.118	*w* = 1/[σ^2^(*F*_o_^2^) + (0.0558*P*)^2^] where *P* = (*F*_o_^2^ + 2*F*_c_^2^)/3
*S* = 1.00	(Δ/σ)_max_ = 0.001
5166 reflections	Δρ_max_ = 0.32 e Å^−^^3^
281 parameters	Δρ_min_ = −0.30 e Å^−^^3^
1 restraint	Absolute structure: Flack (1983), 2490 Friedel pairs
Primary atom site location: structure-invariant direct methods	Flack parameter: 0.01 (2)

### Special details

**Table:**

Geometry. All esds (except the esd in the dihedral angle between two l.s. planes) are estimated using the full covariance matrix. The cell esds are taken into account individually in the estimation of esds in distances, angles and torsion angles; correlations between esds in cell parameters are only used when they are defined by crystal symmetry. An approximate (isotropic) treatment of cell esds is used for estimating esds involving l.s. planes.
Refinement. Refinement of *F*^2^ against ALL reflections. The weighted *R*-factor wR and goodness of fit *S* are based on *F*^2^, conventional *R*-factors *R* are based on *F*, with *F* set to zero for negative *F*^2^. The threshold expression of *F*^2^ > σ(*F*^2^) is used only for calculating *R*-factors(gt) etc. and is not relevant to the choice of reflections for refinement. *R*-factors based on *F*^2^ are statistically about twice as large as those based on *F*, and *R*- factors based on ALL data will be even larger.

### Fractional atomic coordinates and isotropic or equivalent isotropic displacement parameters (Å^2^)

**Table:**

	*x*	*y*	*z*	*U*_iso_*/*U*_eq_	
C1	0.1691 (8)	0.4587 (3)	0.0478 (5)	0.0721 (13)	
H1A	0.0700	0.4306	0.0706	0.087*	
C2	0.2105 (7)	0.5255 (3)	0.0993 (4)	0.0631 (11)	
H2A	0.1359	0.5426	0.1544	0.076*	
C3	0.3610 (6)	0.5673 (2)	0.0701 (4)	0.0520 (10)	
C4	0.4661 (6)	0.5415 (2)	−0.0214 (4)	0.0508 (9)	
C5	0.4159 (7)	0.4721 (3)	−0.0707 (4)	0.0623 (11)	
C6	0.5180 (8)	0.4417 (3)	−0.1549 (4)	0.0762 (14)	
H6A	0.4841	0.3962	−0.1865	0.091*	
C7	0.6682 (8)	0.4797 (3)	−0.1902 (4)	0.0750 (14)	
H7A	0.7391	0.4595	−0.2447	0.090*	
C8	0.7173 (8)	0.5502 (3)	−0.1442 (4)	0.0646 (12)	
C9	0.6189 (7)	0.5800 (2)	−0.0622 (3)	0.0567 (10)	
H9A	0.6521	0.6260	−0.0327	0.068*	
C10	0.9150 (9)	0.6537 (3)	−0.1527 (5)	0.0857 (16)	
H10A	1.0218	0.6701	−0.1901	0.129*	
H10B	0.9624	0.6567	−0.0616	0.129*	
H10C	0.7938	0.6826	−0.1818	0.129*	
C11	0.4201 (6)	0.6379 (2)	0.1375 (3)	0.0508 (9)	
H11A	0.4540	0.6721	0.0781	0.061*	
C12	0.6126 (6)	0.62488 (19)	0.2487 (3)	0.0467 (9)	
H12A	0.7089	0.5959	0.2156	0.056*	
C13	0.5696 (7)	0.5844 (2)	0.3628 (4)	0.0565 (10)	
H13A	0.4229	0.5861	0.3583	0.068*	
H13B	0.6098	0.5351	0.3601	0.068*	
C14	0.6917 (7)	0.6182 (3)	0.4861 (4)	0.0622 (11)	
H14A	0.6783	0.5893	0.5585	0.075*	
C15	0.6009 (9)	0.6922 (3)	0.4946 (5)	0.0815 (14)	
H15A	0.4575	0.6881	0.4976	0.098*	
H15B	0.6774	0.7157	0.5718	0.098*	
C16	0.6143 (8)	0.7356 (3)	0.3786 (5)	0.0770 (14)	
H16A	0.4763	0.7477	0.3285	0.092*	
H16B	0.6894	0.7793	0.4058	0.092*	
C17	0.9372 (7)	0.6747 (3)	0.3784 (4)	0.0672 (12)	
H17A	1.0108	0.7180	0.4108	0.081*	
H17B	1.0137	0.6506	0.3261	0.081*	
C18	0.9227 (7)	0.6269 (3)	0.4897 (4)	0.0667 (12)	
H18A	0.9909	0.6513	0.5689	0.080*	
C19	1.0326 (9)	0.5569 (3)	0.4871 (6)	0.0859 (16)	
H19A	0.9833	0.5274	0.4172	0.103*	
C20	1.1894 (11)	0.5353 (4)	0.5747 (7)	0.126 (3)	
H20A	1.2428	0.5634	0.6459	0.151*	
H20B	1.2493	0.4915	0.5667	0.151*	
N1	0.2684 (6)	0.4343 (2)	−0.0331 (4)	0.0677 (10)	
H1B	0.2353	0.3875	−0.0673	0.081*	
N2	0.7244 (6)	0.69263 (18)	0.2997 (3)	0.0574 (9)	
H2C	0.7373	0.7211	0.2289	0.069*	
O1	0.8655 (5)	0.5813 (2)	−0.1892 (3)	0.0807 (10)	
O2	0.2546 (5)	0.66425 (17)	0.1827 (3)	0.0638 (8)	
H2B	0.2211	0.7051	0.1509	0.096*	
Cl5	0.2012 (3)	0.27667 (8)	−0.07702 (16)	0.1153 (6)	
Cl3	0.8401 (4)	0.35869 (11)	0.53267 (16)	0.1329 (7)	
Cl4	0.6722 (3)	0.24251 (8)	0.26529 (17)	0.1000 (5)	
Cl2	0.3307 (3)	0.37297 (11)	0.3350 (2)	0.1408 (9)	
Fe1	0.64976 (11)	0.35002 (3)	0.33590 (7)	0.0748 (2)	
Cl1	0.7554 (2)	0.42527 (7)	0.21006 (13)	0.0801 (4)	
O3	0.2399 (9)	0.7902 (3)	0.0442 (6)	0.156 (2)	
H3B	0.1900	0.8190	0.0887	0.234*	
H3A	0.1429	0.7743	−0.0162	0.234*	

### Atomic displacement parameters (Å^2^)

**Table:**

	*U*^11^	*U*^22^	*U*^33^	*U*^12^	*U*^13^	*U*^23^
C1	0.073 (3)	0.061 (3)	0.081 (3)	−0.006 (2)	0.017 (3)	−0.003 (3)
C2	0.062 (3)	0.068 (3)	0.064 (3)	0.005 (2)	0.026 (2)	−0.002 (2)
C3	0.056 (2)	0.056 (2)	0.043 (2)	0.0083 (18)	0.0107 (19)	0.0022 (18)
C4	0.056 (2)	0.052 (2)	0.040 (2)	0.0091 (18)	0.0052 (19)	0.0009 (18)
C5	0.070 (3)	0.065 (3)	0.048 (2)	0.010 (2)	0.008 (2)	0.000 (2)
C6	0.089 (4)	0.073 (3)	0.065 (3)	0.011 (3)	0.017 (3)	−0.015 (3)
C7	0.083 (4)	0.094 (4)	0.045 (2)	0.018 (3)	0.012 (3)	−0.015 (2)
C8	0.075 (3)	0.082 (3)	0.038 (2)	0.010 (2)	0.015 (2)	−0.004 (2)
C9	0.068 (3)	0.064 (3)	0.038 (2)	0.005 (2)	0.013 (2)	−0.0013 (19)
C10	0.090 (4)	0.110 (5)	0.063 (3)	−0.019 (3)	0.033 (3)	0.002 (3)
C11	0.060 (3)	0.051 (2)	0.042 (2)	0.0082 (18)	0.0150 (19)	0.0027 (18)
C12	0.059 (2)	0.0405 (19)	0.0438 (19)	0.0040 (16)	0.0192 (18)	−0.0006 (16)
C13	0.056 (2)	0.065 (3)	0.046 (2)	−0.003 (2)	0.0091 (19)	0.010 (2)
C14	0.063 (3)	0.080 (3)	0.044 (2)	0.002 (2)	0.014 (2)	0.009 (2)
C15	0.095 (4)	0.083 (4)	0.073 (3)	0.011 (3)	0.034 (3)	−0.015 (3)
C16	0.094 (4)	0.056 (3)	0.080 (3)	0.010 (2)	0.020 (3)	−0.019 (2)
C17	0.065 (3)	0.073 (3)	0.064 (3)	−0.012 (2)	0.017 (2)	−0.004 (2)
C18	0.063 (3)	0.079 (3)	0.049 (2)	0.002 (2)	−0.003 (2)	−0.010 (2)
C19	0.068 (3)	0.087 (4)	0.094 (4)	0.002 (3)	0.006 (3)	0.001 (3)
C20	0.107 (5)	0.117 (5)	0.140 (6)	0.028 (4)	0.006 (5)	0.027 (5)
N1	0.076 (3)	0.053 (2)	0.071 (2)	−0.0045 (19)	0.012 (2)	−0.006 (2)
N2	0.069 (2)	0.0482 (19)	0.057 (2)	−0.0006 (16)	0.0193 (18)	0.0030 (16)
O1	0.085 (2)	0.109 (3)	0.0543 (18)	−0.005 (2)	0.0304 (17)	−0.0106 (19)
O2	0.0635 (18)	0.0644 (19)	0.0641 (17)	0.0230 (15)	0.0178 (15)	−0.0030 (15)
Cl5	0.1995 (19)	0.0617 (8)	0.1125 (12)	0.0004 (9)	0.0904 (13)	−0.0178 (8)
Cl3	0.201 (2)	0.1015 (12)	0.0886 (10)	0.0237 (14)	0.0246 (11)	0.0161 (10)
Cl4	0.1138 (11)	0.0722 (8)	0.1292 (13)	0.0163 (8)	0.0588 (10)	0.0050 (8)
Cl2	0.1171 (13)	0.1379 (16)	0.203 (2)	0.0635 (11)	0.1064 (14)	0.0824 (15)
Fe1	0.0851 (5)	0.0634 (4)	0.0877 (5)	0.0254 (4)	0.0437 (4)	0.0243 (4)
Cl1	0.0798 (8)	0.0780 (8)	0.0897 (8)	0.0127 (6)	0.0352 (7)	0.0281 (7)
O3	0.163 (5)	0.090 (3)	0.211 (6)	0.004 (3)	0.040 (4)	0.019 (4)

### Geometric parameters (Å, °)

**Table:**

C1—N1	1.314 (6)	C13—H13B	0.9700
C1—C2	1.377 (7)	C14—C15	1.533 (7)
C1—H1A	0.9300	C14—C18	1.543 (7)
C2—C3	1.378 (6)	C14—H14A	0.9800
C2—H2A	0.9300	C15—C16	1.525 (7)
C3—C4	1.442 (6)	C15—H15A	0.9700
C3—C11	1.521 (6)	C15—H15B	0.9700
C4—C9	1.414 (6)	C16—N2	1.503 (6)
C4—C5	1.420 (6)	C16—H16A	0.9700
C5—N1	1.362 (6)	C16—H16B	0.9700
C5—C6	1.397 (6)	C17—N2	1.497 (6)
C6—C7	1.367 (7)	C17—C18	1.531 (7)
C6—H6A	0.9300	C17—H17A	0.9700
C7—C8	1.428 (7)	C17—H17B	0.9700
C7—H7A	0.9300	C18—C19	1.513 (7)
C8—O1	1.346 (6)	C18—H18A	0.9800
C8—C9	1.358 (6)	C19—C20	1.288 (8)
C9—H9A	0.9300	C19—H19A	0.9300
C10—O1	1.435 (7)	C20—H20A	0.9300
C10—H10A	0.9600	C20—H20B	0.9300
C10—H10B	0.9600	N1—H1B	0.9599
C10—H10C	0.9600	N2—H2C	0.9601
C11—O2	1.411 (5)	O2—H2B	0.8499
C11—C12	1.541 (5)	Cl3—Fe1	2.196 (2)
C11—H11A	0.9800	Cl4—Fe1	2.1852 (16)
C12—N2	1.511 (5)	Cl2—Fe1	2.1734 (17)
C12—C13	1.545 (5)	Fe1—Cl1	2.2085 (13)
C12—H12A	0.9800	O3—H3B	0.8501
C13—C14	1.517 (6)	O3—H3A	0.8499
C13—H13A	0.9700		
			
N1—C1—C2	120.6 (5)	C15—C14—C18	108.1 (4)
N1—C1—H1A	119.7	C13—C14—H14A	109.7
C2—C1—H1A	119.7	C15—C14—H14A	109.7
C1—C2—C3	120.8 (4)	C18—C14—H14A	109.7
C1—C2—H2A	119.6	C16—C15—C14	109.2 (4)
C3—C2—H2A	119.6	C16—C15—H15A	109.8
C2—C3—C4	118.7 (4)	C14—C15—H15A	109.8
C2—C3—C11	120.2 (4)	C16—C15—H15B	109.8
C4—C3—C11	121.0 (4)	C14—C15—H15B	109.8
C9—C4—C5	118.3 (4)	H15A—C15—H15B	108.3
C9—C4—C3	124.2 (4)	N2—C16—C15	108.9 (4)
C5—C4—C3	117.5 (4)	N2—C16—H16A	109.9
N1—C5—C6	119.7 (5)	C15—C16—H16A	109.9
N1—C5—C4	119.2 (4)	N2—C16—H16B	109.9
C6—C5—C4	121.1 (5)	C15—C16—H16B	109.9
C7—C6—C5	119.1 (5)	H16A—C16—H16B	108.3
C7—C6—H6A	120.4	N2—C17—C18	109.9 (4)
C5—C6—H6A	120.4	N2—C17—H17A	109.7
C6—C7—C8	120.6 (4)	C18—C17—H17A	109.7
C6—C7—H7A	119.7	N2—C17—H17B	109.7
C8—C7—H7A	119.7	C18—C17—H17B	109.7
O1—C8—C9	125.8 (5)	H17A—C17—H17B	108.2
O1—C8—C7	113.7 (4)	C19—C18—C17	111.6 (4)
C9—C8—C7	120.5 (5)	C19—C18—C14	113.2 (4)
C8—C9—C4	120.3 (4)	C17—C18—C14	108.0 (4)
C8—C9—H9A	119.8	C19—C18—H18A	107.9
C4—C9—H9A	119.8	C17—C18—H18A	107.9
O1—C10—H10A	109.5	C14—C18—H18A	107.9
O1—C10—H10B	109.5	C20—C19—C18	124.7 (6)
H10A—C10—H10B	109.5	C20—C19—H19A	117.7
O1—C10—H10C	109.5	C18—C19—H19A	117.7
H10A—C10—H10C	109.5	C19—C20—H20A	120.0
H10B—C10—H10C	109.5	C19—C20—H20B	120.0
O2—C11—C3	110.2 (4)	H20A—C20—H20B	120.0
O2—C11—C12	110.7 (3)	C1—N1—C5	123.2 (4)
C3—C11—C12	107.4 (3)	C1—N1—H1B	118.4
O2—C11—H11A	109.5	C5—N1—H1B	118.4
C3—C11—H11A	109.5	C17—N2—C16	109.1 (3)
C12—C11—H11A	109.5	C17—N2—C12	109.1 (3)
N2—C12—C11	112.8 (3)	C16—N2—C12	113.4 (4)
N2—C12—C13	107.4 (3)	C17—N2—H2C	108.4
C11—C12—C13	114.7 (3)	C16—N2—H2C	108.4
N2—C12—H12A	107.2	C12—N2—H2C	108.4
C11—C12—H12A	107.2	C8—O1—C10	116.8 (4)
C13—C12—H12A	107.2	C11—O2—H2B	109.0
C14—C13—C12	109.5 (3)	Cl2—Fe1—Cl4	109.81 (9)
C14—C13—H13A	109.8	Cl2—Fe1—Cl3	108.14 (9)
C12—C13—H13A	109.8	Cl4—Fe1—Cl3	109.65 (7)
C14—C13—H13B	109.8	Cl2—Fe1—Cl1	109.82 (6)
C12—C13—H13B	109.8	Cl4—Fe1—Cl1	108.41 (6)
H13A—C13—H13B	108.2	Cl3—Fe1—Cl1	111.00 (8)
C13—C14—C15	107.7 (4)	H3B—O3—H3A	109.5
C13—C14—C18	112.0 (3)		

### Hydrogen-bond geometry (Å, °)

**Table:**

*D*—H···*A*	*D*—H	H···*A*	*D*···*A*	*D*—H···*A*
N1—H1B···Cl5	0.96	2.10	3.023 (4)	161
N2—H2C···Cl5^i^	0.96	2.08	3.039 (4)	173
O2—H2B···O3	0.85	2.00	2.799 (6)	156
O3—H3B···Cl5^ii^	0.85	2.71	3.070 (6)	108

Symmetry codes: (i) −*x*+1, *y*+1/2, −*z*; (ii) −*x*, *y*+1/2, −*z*.
